# Power signatures of habenular neuronal signals in patients with bipolar or unipolar depressive disorders correlate with their disease severity

**DOI:** 10.1038/s41398-022-01830-3

**Published:** 2022-02-22

**Authors:** Saurabh Sonkusare, Qiong Ding, Yingying Zhang, Linbin Wang, Hengfen Gong, Alekhya Mandali, Luis Manssuer, Yi-Jie Zhao, Yixin Pan, Chencheng Zhang, Dianyou Li, Bomin Sun, Valerie Voon

**Affiliations:** 1 Department of Psychiatry, University of Cambridge, Cambridge, United Kingdom; 2 Department of Neurosurgery, Center for Functional Neurosurgery, Ruijin Hospital, Shanghai Jiao Tong University School of Medicine, Shanghai, China; 3 Institute of Science and Technology for Brain-Inspired Intelligence, Fudan University, Shanghai, China; 4 Shanghai Pudong New Area Mental Health Center, Tongji University School of Medicine, Shanghai, China; 5 Key Laboratory of Computational Neuroscience and Brain-Inspired Intelligence, Fudan University, Ministry of Education, Shanghai, China

**Keywords:** Diagnostic markers, Neuroscience

## Abstract

The habenula is an epithalamic structure implicated in negative reward mechanisms and plays a downstream modulatory role in regulation of dopaminergic and serotonergic functions. Human and animal studies show its hyperactivity in depression which is curtailed by the antidepressant response of ketamine. Deep brain stimulation of habenula (DBS) for major depression have also shown promising results. However, direct neuronal activity of habenula in human studies have rarely been reported. Here, in a cross-sectional design, we acquired both spontaneous resting state and emotional task-induced neuronal recordings from habenula from treatment resistant depressed patients undergoing DBS surgery. We first characterise the aperiodic component (1/f slope) of the power spectrum, interpreted to signify excitation-inhibition balance, in resting and task state. This aperiodicity for left habenula correlated between rest and task and which was significantly positively correlated with depression severity. Time-frequency responses to the emotional picture viewing task show condition differences in beta and gamma frequencies for left habenula and alpha for right habenula. Notably, alpha activity for right habenula was negatively correlated with depression severity. Overall, from direct habenular recordings, we thus show findings convergent with depression models of aberrant excitatory glutamatergic output of the habenula driving inhibition of monoaminergic systems.

## Introduction

Major depressive disorder is a major public health issue representing the greatest global burden of disability [1]. Yet, many remain treatment-refractory [2]. Deep brain stimulation (DBS) holds promise for treating resistant depression with potential targeting of brain structures such as the subcallosal cingulate cortex (scCing), anterior limb of the internal capsule [3] and medial forebrain bundle [4, 5]. Another brain region, the habenula, a small evolutionarily conserved epithalamic structure, is also a plausible DBS target with two previous pilot clinical case studies demonstrating its therapeutic utility with DBS [6, 7].

**Fig. 1:**
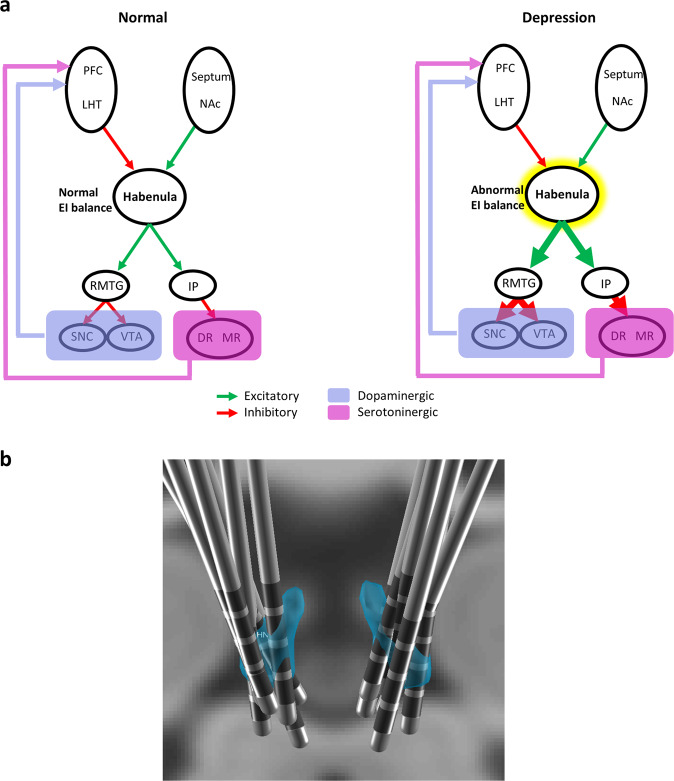
Habenula circuitry and electrode localisation. **a** left- simplistic illustration of normal excitatory and inhibitory connections of habenula and their downstream neurotransmitter profile, right—hyperactive habenula in depression with increased excitation-inhibition (EI) balance leading to greater downstream effects (shown in thickened arrows). For detailed connections of the habenula see [13, 74–76]. PFC—prefrontal cortex, LHT—lateral hypothalamus; NAc—nucleus accumbens; RMTG—rostromedial tegmental region; IP—interpeduncular nucleus; SNC—substantia nigra pars compacta; VTA—ventral tegmental area; DR- dorsal raphe nuclei, MR—medial raphe nuclei **b** electrode locations reconstructed using Lead-DBS with habenula represented in blue.

The habenula is a key hub connecting the midbrain and the prefrontal cortex [8–10] and acts as a major node in the reward signal pathway [10]. It is believed to encode negative reward mechanisms such as negative prediction error signals or unexpected aversive or loss events by driving inhibition onto dopaminergic neurons [11, 12]. It further regulates downstream serotonergic raphe neurons [13] and relays a powerful inhibitory influence on downstream midbrain structures which include both the dopaminergic and serotonergic systems [14, 15] (Fig. 1a).

Dysfunctional habenular activity has been associated with major depression [16] with convergent evidence of its hyperactivity in depression coming from human and animal studies. Its pathological hyperactivity in rodent models of depression is believed to be related to increased burst firing [17] and remarkably ketamine, a rapidly acting antidepressant, blocks this burst firing [17, 18]. Burst firing refers to the irregular episodes of bursts of neuronal spikes followed by inactivity [19] and is associated with neuronal noise [20]. Burst firing has been linked to the non-oscillatory or the aperiodic component—1/f like properties—of the neuronal signal [21]. This component is the often neglected but has recently garnered renewed interest. For instance, cognitive and perceptual states alter this property and it has been shown to be a potential biological marker in development and aging [22, 23] and in disease states such as attention-deficit hyperactivity disorder [24] and schizophrenia [25]. Furthermore, aperiodic component has recently been interpreted as an index of excitation-inhibition balance (E/I) [26]. This is particularly relevant for habenular activity as it is one of the few structures in the brain modulating both dopaminergic and serotoninergic activity with complex upstream and downstream excitatory and inhibitory effects. As such quantifying this aperiodic component may provide insights on its role in depression. However, human neuronal recordings from habenula have rarely been investigated and not to our knowledge for aperiodic power signatures.

Here, we acquired neuronal recordings from bilateral habenula of treatment resistant depressed patients undergoing DBS in resting state and an emotional picture viewing task. We evaluated the aperiodic non-oscillatory activity and task evoked activity. Given habenular hyperactivity reported in depression, we hypothesised the aperiodic exponent to be associated with depression severity. Lack of prior habenular neuronal recordings from humans precluded a specific hypothesis about the evoked activity. However, its role in emotional valence led us to anticipate differences in task evoked responses to positive and negative affective images [27].

## Materials and methods

### Participants

This study was registered as a clinical trial (NCT03347487). The patients underwent DBS surgery for refractory depression targeting the habenula at Ruijin Hospital, Shanghai Jiao Tong University. All the patients either had bipolar depression or unipolar depression. Patient demographics, medication use and diagnoses are presented in Table 1. Neuropsychological tests including Hamilton Depression Rating Scale (HAMD) were administered one week prior to the surgery. The clinical outcomes of all the patients in the trial (which also included patients with schizophrenia and chronic pain) are under preparation in a separate manuscript. The ethics committee of Ruijin Hospital, Shanghai Jiao Tong University School of Medicine, approved all procedures used in this study. All patients provided written informed consent in accordance with the Declaration of Helsinki.

**Table 1:** Patient profile.

ID	Sex	Age (yrs)	Handed –ness R-right	Marital Status	Medications	Tobacco/ Alcohol (y-yes n-no)	Diagnosis	HAMD
P1	M	46	R	divorced	Lamotrigine-100mg/d; Seroquel-100mg/d; Magnesium Valproate-0.75 g/d,; Amfebutamone-0.3 g/d,; Clonazepam-16-20mg/d	y/y	Bipolar Disorder; Substance Dependence-(Clonazepam, Alcohol); Gambling Disorder	23
P2	M	21	R	single	Olanzapine-5mg/d, Mirtazapine-15mg/d	n/n	Depression (Unipolar)	24
P3	F	30	R	single	Olanzapine-5mg/d, Escitalopram Oxalate-20mg/d, Mirtazapine-15mg/d, Lithium Carbonate-0.9 g/d	y/y	Bipolar Disorder	21
P4	M	48	R	married	Sodium Valproate Sustained-release Tablets-1g/d, Clonazepam-0.5 mg/d	y/y	Bipolar Disorder	23
P5	F	28	R	single	Venlafaxine-225mg/d, Mirtazapine-30mg/d, Lithium Carbonate-0.9 g/d, Pregabalin-225mg/d, Zopiclone-7.5 mg/d	y/n	Depression (Unipolar) Or Bipolar II	36
P6	M	48	R	divorced	Duloxetine-60mg, Olanzapine-5mg, Clonazepam-1mg-1mg, Tandospirone citrate-20mg	y/n	Bipolar Disorder II, Generalised Anxiety Disorder	39

*HAMD* Hamilton Depression Rating Scale.

### Surgical procedure

Quadripolar DBS electrodes (model 3389; contact: 1.5 mm, distance: 0.5 mm, diameter: 1.27 mm; Medtronic, Minneapolis, MN, USA) were implanted under general anaesthesia bilaterally using MRI-guided targeting (3.0 T, General Electric, The MRI was co-registered with a CT image (General Electric, 314 Waukesha, WI, USA) with the Leksell stereotactic frame to obtain the coordinate values [7, 28]. The electrode leads were temporarily externalised during which physiological recordings were acquired.

### Habenula localisation and electrode mapping

A stereotactic frame was used to place the electrodes on the bilateral habenula. Susceptibility-weighted imaging (SWI) and quantitative susceptibility mapping (QSM) in 3.0-T MRI have been demonstrated to better localise small subthalamic and epithalamic nuclei [29]. Here, we used SWI and QSM imaging to localise the habenula which followed identical procedures established previously [29, 30].

Post-operative CT and pre-operative T1 MRI were used to reconstruct the electrode trajectories and their locations by employing the LEAD-DBS toolbox [31] (Fig. 1). Briefly. a two-stage linear registration as implemented in Using Advanced Normalization Tools (ANT) [32] was used and the post-operative CT co-registered to pre-operative MRI and spatially normalised into MNI_ICBM_2009b_NLIN_ASYM space [33]. Pacers algorithm [34] was used to localise electrodes in MNI space.

### Data preprocessing

The LFP and electroencephalogram (EEG) data were recorded using a BrainAmp MR amplifier (Brain Products, Gilching, Germany) with a 500 Hz sample rate and a notch filter set at 50 Hz to remove the power line noise interference. The left mastoid was used as the reference electrode. Scalp EEG was recorded from 7 frontal electrodes (Fp1, Fp2, F3, F4, F7, F8, Fz) using the 10-20 placement system. LFP data were collected from all contacts on both electrodes (L0-L3 and R0-R3).

Using LEAD DBS toolbox we calculated the contacts that either were within the habenula or the closest contacts and which were visually confirmed. We used bipolar re-referencing to assign the anode to the contact within or closest to the habenula and the cathode as the next closest (e.g. R0-R1, R1-R2, or R2-R3). Bipolar referencing nullifies the volume conducted activity from regions distal to the contacts of interest thus providing spatially resolved signal. From the 12 bilateral electrodes (48 contacts), 12 contacts showed clear localisation within the habenula with 8 contacts from 12 electrodes selected as the anode. The other 4 contacts selected showed close proximity (0.1, 0.5, 1.8, 3 mm).

The pre-processing steps undertaken for the LFP and EEG data were performed using EEGLAB [35] and custom routines programed in MATLAB (MathWorks, MA, USA). Specifically, these included band pass filtering and visual inspection for trial rejection. For rest data, data was segmented into 5 s windows. After rejecting trials with artefacts, 12 segments corresponding to 60 s were used for analysis. The scalp EEG data was bandpass filtered from 0.5–45 Hz. An independent components analysis was run on the scalp EEG recordings to identify and remove the artifacts associated with blinks and lateral eye-movements. For the task-based analysis, each trial was then epoched by time-locking activity to the onset of the affective pictures.

### Electrode recordings

Electrodes within habenula or closest to it were used for bipolar re-referencing. Local field potentials (LFPs) were recorded from bilateral habenula during both resting state and an emotional task. For the resting state data acquisition, patients viewed a fixation cross on a computer screen for approximately 2–3 min. The emotional picture viewing task has been described in detail in a previous publication [27]. Briefly, 90 different picture stimuli from the validated International Affective Picture System [36] were shown (30 in each valence condition-positive, neutral, negative) for 2 s, with 15 images (5/category) rated for valence and 15 images (5/category) for arousal using a sliding scale (0–100) (Supplementary Table 1).

### Power profile

Power spectra for resting state data were computed using *pwelch* function in Matlab with a window of 3 s, default overlap (8 segments with 50% overlap). For task data window of 1.25 s was used with default overlap (8 segments with 50% overlap). Specifically, the aperiodic component quantifies the slope of the power decrease [21] and its quantification is often hampered by variability of the power peaks seen in the data. Here we used Fitting Oscillations and One-Over-F (FOOOF) algorithm [21] to accurately (applied to 1–36 Hz power spectra) separate the LFP power spectral densities into aperiodic (1/f-like component) and periodic oscillatory components modelled as Gaussian peaks. The model was fitted for individual subject trials and averaged across trials

The settings for the algorithm were set as: peak_width_limits = [0.5, 12]; min_peak_height = 0; max_n_peaks = 12; peak_threshold = 2; aperiodic_mode = “fixed”; and verbos = ‘True’. The model fit was assessed by goodness of fit as estimated by R^2^ values. For resting data, the group mean R^2^ values for left habenula power spectral fit was 0.92 and that for the right habenula power spectral fit was 0.91. For task data the group mean R^2^ values for left habenula power spectral fit was 0.90 and that for the right habenula power spectral fit was 0.93. For task data all clean preprocessed trials from the three conditions were concatenated for each subject and the power profile and aperiodic component computed for each subject.

### Task evoked activity

Event related spectral perturbation (ERSP) measures variations in amplitude of the broad band EEG frequency spectrum induced by an experimental event relative to the baseline [37]. We computed individual trial ERSPs as they have been demonstrated to be robust to outlier trial activity [38]. We used a 500 millisecond baseline (fixation cross presentation) and post-stimulus window of 1500 ms. Fast-fourier transform (fft) was employed to compute ERSPs using the EEGLAB toolbox [35]. The changes in ERSP may correspond to a narrow-band of event-related desynchronisation (ERD) or synchronisation (ERS) or power spectrum reduction or increase respectively. All the ERSPs for each condition across subjects were concatenated which were used for further statistical analyses.

### Statistical testing

Paired *t*-tests were used for comparisons of differences. Pearson’s correlation was used for strength of association between two variables. Bonferroni correction was employed for multiple comparison wherever applicable. To compare time frequency maps (ERSPs) between conditions, we used non-parametric cluster-based permutation [39] to identify significant clusters of power differences (time window and frequency band) induced by emotional images. 1000 iterations of permutations were performed, and null distribution generated with suprathreshold-clusters significance detected at *P* < 0.01. We restricted cluster size >200 to find robust differences and avoiding non-specific small clusters given the small sample size. Average power in the determined frequency band for ERSPs identified by cluster-based permutation between conditions were further compared using post-hoc paired t-tests with Bonferroni correction. Significant cluster means were then correlated with depression scores.

## Results

### Patient demographics

Six patients (2 females, mean age: 36.8 years) with refractory depression were included. Patient demographics are reported in Table 1.

### Aperiodic component – 1/f

**Fig. 2:**
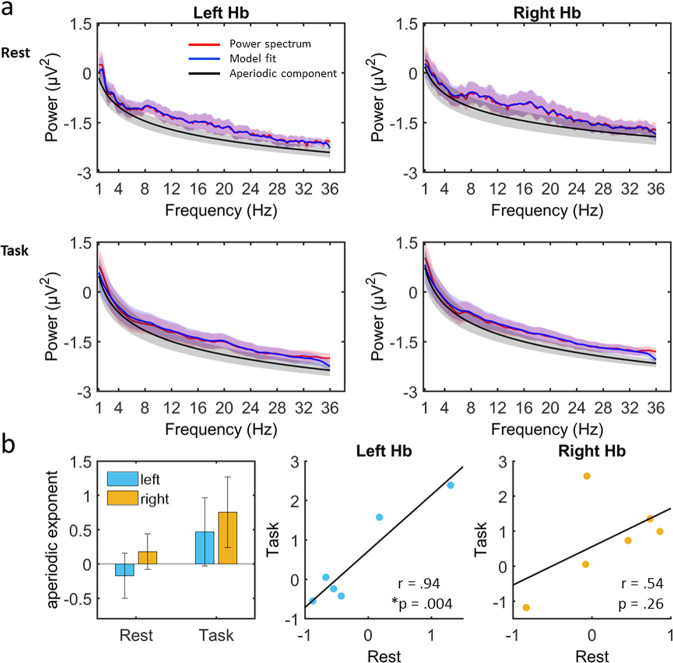
Power profile of habenular recordings. **a** Power profile for resting state (top) and task (bottom) shown in red. Power fit via *fooof* algorithm is shown in blue and aperiodic component shown in black. Shading indicates standard error of mean. Note the overlapping colour and SEM shading of the power spectrum, the model fit to the power spectrum and the aperiodic component. **b** The bar plot (left) shows the aperiodic component with no significant differences between left (blue) and right habenula (yellow) with greater left habenula aperiodic component in task relative to rest (Error bars indicate stand error of mean). The scatter plots of the aperiodic components between rest and task shows a significant positive correlation for left habenula (blue) but not for right habenula (yellow). Bonferroni correction *P < 0.025.

For resting data, the group mean R^2^ values for left habenula power spectral fit was 0.92 and that for the right habenula power spectral fit was 0.91. For task data the group mean R^2^ values for left habenula power spectral fit was 0.90 and that for the right habenula power spectral fit was 0.93. The rest and task data showed similar power profiles for left and right habenula (Fig. 2). Individual patient’s spectrum are shown in supplementary (Supplementary Fig. 1). On an exploratory basis, we assessed the relationship between aperiodic components of rest and task. Left habenula seemed to show increased aperiodic component in task when compared to rest (mean = −0.17, SD = 0.81; *t(5)=−2.97, P* = *0.03*) which however did not survive multiple comparison (Bonferroni significance *P* < 0.013). Right habenula activity did not show significant difference (mean = 0.18, SD = 0.63; *t(5)=−1.32, P* = *0.24*) (Fig. 2b left). The aperiodic component values were significantly correlated between rest and task data for the left habenula (*r* = *0.94, P* = *0.004*) (Bonferroni significance *P* < *0.025*) but not for right habenula (*r* = *0.54, P* = *0.26*) (Fig. 2b, middle and right panel).

### Aperiodic component’s relationship with depression severity

**Fig. 3:**
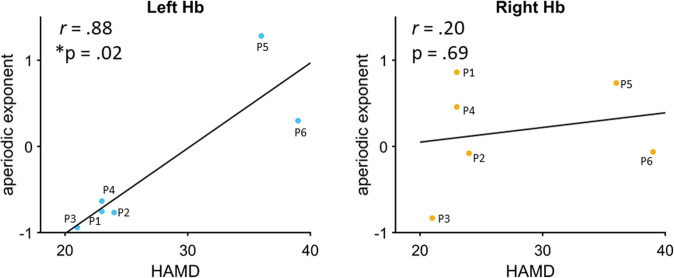
Relationship of aperiodic component with depression scores. The aperiodic component of left habenula (Hb) (blue) showed a positive correlation with baseline depression scores measured using the Hamilton depression rating scale (HAMD) with no significant relationship observed for the right habenula (yellow). Bonferroni correction *P < 0.025.

In our main hypothesis, we then correlated the aperiodic component with the depression severity. The left habenula aperiodic component was significantly positively correlated with depression severity (*r* = *0.88, P* = *0.015*) (Bonferroni significance *P* < *0.025*) (Fig. 3 left) but not the right habenula (*r* = *0.20, P* = *0.69*) (Fig. 3 right). We also replicate this result when cut-off frequency range for estimation of aperiodic component was 1–40 Hz (Supplementary Fig. 2).

### Task evoked activity

The left and right habenula showed early stimulus locked responses to the affective images with increased theta and alpha frequency activity (Supplementary Fig. 3). The left and right habenula across all conditions showed ERD in alpha and beta frequencies (Supplemenatry Fig. 3). Left habenula showed differences in lower beta and gamma ERD for negative compared to positive condition (beta: Positive mean = −0.38, SD = 0.73; Negative mean = −3.73, SD = 1.81, *t(5)=6.92, P* = *0.0009*; gamma: Positive mean = −0.27, SD = 0.21; Negative mean = −1.77, SD = 1.05, *t(5)=4.20, P* = *0.008*). Right habenula showed alpha ERD at ~1 s which was greater for negative than positive stimuli (Positive mean = −0.17, SD = 0.27; Negative mean = −3.30, SD = 0.89, *t(5)=8.64, P* = *0.0003*). Bonferroni significance at *P* < .017.

### Alpha ERD relationship with depression severity

**Fig. 4:**
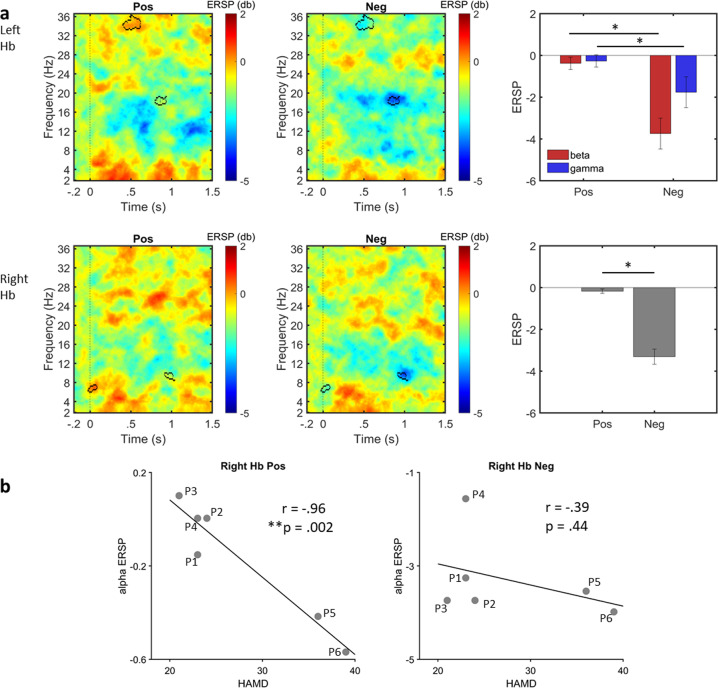
Task induced activity and relationship with depression scores. **a** Event related spectral perturbation (ERSP) maps. Top panel: left habenula; bottom panel: right habenula. Significant cluster bounded by black outline tested with permutation testing. Group mean of the significant ERSP clusters showing significant differences between valence conditions on t-tests. Errors bars indicate stand error of mean. Bonferroni correction *P < *.017*. Pos—positive, Neg—negative. **b** Left: significant negative correlation of significant alpha cluster of right habenula activity for positive stimuli and Hamilton Depression rating scale (HAMD) i.e., greater depressive symptoms associated with greater habenula alpha event-related desynchronization (ERD) (or decrease in alpha power) in response to pleasant stimuli; right: no significant relationship was observed between the alpha ERSP significant cluster with HAMD scores for negative stimuli. Bonferroni correction at **P < *0.0083* (correlation coefficient of gamma and beta cluster for left habenula activity was also tested and thus multiple correction applied for 6 tests considering beta and gamma clusters for left habenula activity, see Supplementary Fig. 2).

Two significant clusters n the alpha range were apparent. However the cluster immediately at the stimulus onset is unlikely of interest given such an early latency. Hence we focussed only on the late alpha cluster ~at 1.5 s post stimuli. Furthermore, alpha ERD during this latency range has also been shown to be emotionally relevant in STN studies [27, 40, 41]. The significant alpha ERSP cluster for positive stimuli in the right habenula showed a negative correlation with depression scores (*r* = *−0.96, P* = *0.002*) with Bonferroni significance *P* < *0.008* (Fig. 4b). The alpha ERD for negative stimuli did not show any significant association (*r* = *−0.39, P* = *0.44*) (Fig. 4b). Beta and gamma ERSP significant clusters in the right and left habenula were unrelated to depression severity (Supplementary Fig. 4).

## Discussion

We characterised the power signatures of human habenular recordings demonstrating that the non-oscillatory aperiodic component of the left habenula predicts depression severity. Emotional task induced responses showed significant differences in alpha ERD in right habenula, late beta ERD and early gamma ERD for left habenula. Furthermore, the alpha ERD for positive stimuli in right habenula negatively correlated with depression severity.

### Habenular aperiodic exponent and excitatory-inhibitory (E/I) balance

The aperiodic exponent has been interpreted as signifying E/I balance [26, 42]. It has been related to the integration of the underlying synaptic currents, which gives rise to the 1/f-like nature of the power spectral density (PSD). Disruptions in the E/I balance (i.e., greater excitatory relative to inhibitory synaptic currents, or vice versa) can lead to abnormal downstream effects on a meso- and macroscale network level. E/I balance is also not a static property, but changes depending on behavioural state [43] and task demands [44] suggesting that this property is under fine dynamic control. This is consistent with rapid adaptive properties of the brain structures and their upstream/downstream effects on global brain networks [45, 46]. Our results from aperiodic characterisation shows association between rest and task for left habenula only. Although asymmetry of the habenula structure and function have been reported [47], further studies are warranted to uncover the laterality differences in power signatures.

E/I imbalance is proposed to be underlying the pathophysiology of various neuropsychiatric disorders [48]. Abnormal E/I balance may be caused by habenular hyperactivity in depression and its increased burst firing has been reported in human [49] and animal studies [17]. Critically, in our study, the greater E/I ratio of left habenula, as indexed by the aperiodic exponent, correlated with higher baseline depression severity thus presumably reflecting the increased excitatory habenular dysfunction in major depression. The lateral habenula projects glutamatergic excitatory input to the rostromedial tegmentum which then acts via GABA-ergic inhibitory inputs to dopaminergic and serotonergic midbrain nuclei [14, 15]. Excessive neuronal firing can potentially strengthen lateral habenula output in turn causing greater suppression of dopaminergic and serotonergic projections leading to depression [50]. Importantly our results are convergent with evidence from rodent models of depression which emphasize a bursting mode of firing in the lateral habenula [17] which was specifically targeted by the rapid antidepressant effects of ketamine [17, 18]. Although clinical trials of ketamine in humans have been undertaken [51, 52], our results give potential insight on its effect on neuronal properties of aperiodicity thus indicating further studies.

Habenular efferents play a prominent role in reducing brain serotonin activity associated with depressive behaviour and have led to the proposition that functional inactivation of the habenula by deep brain stimulation may be effective treatment for refractory depression [6, 53]. It is possible that DBS at clinical frequency of 130 Hz restores the aperiodic properties of habenular neurons to normal levels which future studies can investigate. However, support for this notion comes from animal studies showing habenular stimulation at non-clinical frequency inhibits serotonin neurons in the dorsal raphe [54–57]. Furthermore, enhanced habenula metabolism and reduced brain serotonin levels have been observed in animal depression models and can be attenuated by antidepressants [50] or lateral habenular lesions [17]. Lastly, patients experiencing transient depressive episodes elicited by tryptophan depletion show increased habenular cerebral blood correlating with depression scores [49]. DBS of habenula in major depression is thus grounded on multiple convergent lines of evidence.

We did not see robust state dependent changes of aperiodic properties in our data, which other studies have demonstrated [58, 59]. Further research on investigating the aperiodic component with various modalities will be useful to uncover its neurobiological underpinnings and its utility. Although the aperiodic component did not differ significantly between rest and task, overall, power spectrum observed with rest and task deserve some remarks especially a smoother spectrum observed with the task data. Resting state is a free behavioural paradigm reflecting spontaneous neuronal activity which may result in a more complex power spectrum. In contrast task evoked activity constrains the spontaneous activity by increased attentional demands. For instance, alpha activity is often reduced when attentional demands are high [60, 61]. It is also possible that different parameters for estimating power may result in smoother power spectrum of task data. However, we show consistent results of association of aperiodic component with depression severity with 3 sec epochs (matching that of task data) of resting data (Supplementary Fig. 5).

### Habenular emotional processing and alpha activity

Our results also show frequency modulations in the emotional viewing task. Specifically, for the right habenula, alpha ERD was increased for negative pictures and decreased for positive pictures. In contrast, beta ERD in the left habenula was greater for negative than positive stimuli. A decrease in alpha activity to both positive and negative stimuli is commonly observed with intracranial recordings from subthalamic nucleus (STN) in patients with Parkinson’s disease implicated in the subjective evaluation of emotional valence [27, 62, 63]. Alpha and beta band activity is also decreased in the subcallosal cingulate to both positive and negative pictures in depressed patients undergoing DBS [40]. The time window of the relatively late (750–2500 ms) habenular ERD in alpha and beta (Fig 5) is similar to the time window reported in previous M/EEG studies on emotional processing [64, 65]. Kuhn et al. have also suggested the higher sensitivity of the scCing for negative stimuli may represent a negativity bias in major depression [66]. Thus, our results are consistent with the pattern of responses of mid brain structures in emotional picture viewing task.

Lastly, we show that patients with greater depressive symptoms had greater task-induced alpha-ERD response to positive stimuli. This pattern is opposite to that found in Parkinson’s disease patients in whom greater depression was associated with reduced STN alpha-ERD response to positive and greater alpha-ERD response to negative emotional images [41]. Habenula neurons have inhibitory effects on dopaminergic neurons [10, 13] encoding reward prediction error in the opposite direction to that of dopaminergic neurons [13]. Thus, a plausible explanation of the differing habenular and STN observations may be that greater habenula ERD to pleasant stimuli reflects a deficit of processing positive information with greater depression severity characterised by lower dopaminergic activity. This may resemble the clinical symptom of anhedonia, which is common in depression and may also be related to our patient cohort characterised by high severity of depression. Major depression might result in less variability in processing of negative stimuli but greater variability for positive stimuli as a function of depression severity. Alpha ERD may thus be a potential biomarker tracking depression severity across a range of neural nodes and monitoring of treatment response.

We have recently shown that stimulation at alpha frequency targeting the STN can shift subjective negative emotional biases suggesting a strong link between alpha desynchronization and subjective emotional processing [27]. Here, we also confirm a critical role for alpha activity in emotional processing within the habenula. Furthermore, we show alpha coherence in this frequency range between left and right habenula and the prefrontal cortex (Supplementary Fig. 6). As a connecting node between two major sub-systems, we suggest alpha frequency band as a key mediator in emotional processing linking habenula to prefrontal cortical function. The only study which investigated habenular recordings in emotional task found increased alpha-theta activity for negative stimuli and prefrontal connectivity (assessed with MEG) [28]. Notably, our study only assessed depressed patients whereas Huang et al. assessed depression, schizophrenia and pain patients [28] which may account for differences between the two studies. Nonetheless, further studies targeting habenular stimulation at lower frequencies may open newer avenues for modulating emotional behaviours.

Our study is not without limitations. First, our severe resistant depression cohort may limit generalisability and our sample size is also limited. However human habenular recordings have only been investigated once [57]. Second, specific psychiatric diagnosis and additional co-morbidities, varied medications and their interactions may affect E/I ratio and consequently the aperiodic component. Third, all patients in this study barring one had bipolar depression and hence it was not possible to investigate if the aperiodic component can differentiate the depression subtypes. Fourth, 1/f like properties are commonly reported with macroscale EEG/MEG recordings [67–71] but which have also been demonstrated at microscale [72] and mesoscale [73]. However, it is possible that the subtle varied cytoarchitectural profiles of different regions in the cortex and the sub-cortex might affect 1\f properties. Future studies with recordings at micro, meso and macroscale can clarify these issues. Finally given the small size of the habenula and challenges associated with localising smaller structures, our study is unable to discriminate between the medial and lateral habenula.

In summary, our data extends current knowledge on habenula function in depression through direct habenular neuronal recordings. Habenula power signatures and alpha ERD activity in task may act as potential biomarkers across an emotional neural network, and a target for neuromodulation.

## Supplementary information

Supplementary Material

## References

[1] World Health Organization, Depression and other common mental disorders: global health estimates. 2017.

[2] Schlaepfer TE, Lieb K (2005). Deep brain stimulation for treatment of refractory depression. Lancet.

[3] Denys D, Graat I, Mocking R, de Koning P, Vulink N, Figee M (2020). Efficacy of deep brain stimulation of the ventral anterior limb of the internal capsule for refractory obsessive-compulsive disorder: a clinical cohort of 70 patients. Am J Psychiatry.

[4] Delaloye S, Holtzheimer PE (2014). Deep brain stimulation in the treatment of depression. Dialogues Clin Neurosci.

[5] Coenen VA, Bewernick BH, Kayser S, Kilian H, Boström J, Greschus S, (2019). Superolateral medial forebrain bundle deep brain stimulation in major depression: a gateway trial. Neuropsychopharmacology.

[6] Sartorius A, Kiening KL, Kirsch P, von Gall CC, Haberkorn U, Unterberg AW (2010). Remission of major depression under deep brain stimulation of the lateral habenula in a therapy-refractory patient. Biol Psychiatry.

[7] Zhang C, Kim S-G, Li D, Zhang Y, Li Y, Husch A (2019). Habenula deep brain stimulation for refractory bipolar disorder. Brain Stimul.

[8] Salas R, Baldwin P, de Biasi M, Montague PR (2010). BOLD responses to negative reward prediction errors in human habenula. Front Hum Neurosci.

[9] Shabel SJ, Proulx CD, Trias A, Murphy RT, Malinow R (2012). Input to the lateral habenula from the basal ganglia is excitatory, aversive, and suppressed by serotonin. Neuron.

[10] Matsumoto M, Hikosaka O (2007). Lateral habenula as a source of negative reward signals in dopamine neurons. Nature.

[11] Lawson RP, Seymour B, Loh E, Lutti A, Dolan RJ, Dayan P (2014). The habenula encodes negative motivational value associated with primary punishment in humans. Proc Natl Acad Sci USA.

[12] Matsumoto M, Hikosaka O (2009). Representation of negative motivational value in the primate lateral habenula. Nat Neurosci.

[13] Hu H, Cui Y, Yang Y (2020). Circuits and functions of the lateral habenula in health and in disease. Nat Rev Neurosci.

[14] Balcita‐Pedicino JJ, Omelchenko N, Bell R, Sesack SR (2011). The inhibitory influence of the lateral habenula on midbrain dopamine cells: ultrastructural evidence for indirect mediation via the rostromedial mesopontine tegmental nucleus. J Comp Neurol.

[15] Jhou TC, Fields HL, Baxter MG, Saper CB, Holland PC (2009). The rostromedial tegmental nucleus (RMTg), a GABAergic afferent to midbrain dopamine neurons, encodes aversive stimuli and inhibits motor responses. Neuron.

[16] Boulos L-J, Darcq E, Kieffer BL (2017). Translating the habenula—from rodents to humans. Biol Psychiatry.

[17] Yang Y, Cui Y, Sang K, Dong Y, Ni Z, Ma S (2018). Ketamine blocks bursting in the lateral habenula to rapidly relieve depression. Nature.

[18] Zanos P, Gould TD (2018). Mechanisms of ketamine action as an antidepressant. Mol Psychiatry.

[19] Izhikevich EM (2000). Neural excitability, spiking and bursting. Int J Bifurc Chaos.

[20] Ozer M, Perc M, Uzuntarla M (2009). Controlling the spontaneous spiking regularity via channel blocking on Newman-Watts networks of Hodgkin-Huxley neurons. EPL (Europhys Lett).

[21] Donoghue T, Haller M, Peterson EJ, Varma P, Sebastian P, Gao R (2020). Parameterizing neural power spectra into periodic and aperiodic components. Nat Neurosci.

[22] Gao R, van den Brink RL, Pfeffer T, Voytek B (2020). Neuronal timescales are functionally dynamic and shaped by cortical microarchitecture. Elife.

[23] Tran TT, Rolle CE, Gazzaley A, Voytek B (2020). Linked sources of neural noise contribute to age-related cognitive decline. J Cogn Neurosci.

[24] Robertson MM, Furlong S, Voytek B, Donoghue T, Boettiger CA, Sheridan MA (2019). EEG power spectral slope differs by ADHD status and stimulant medication exposure in early childhood. J Neurophysiol.

[25] Ramsay I, Lynn P, Lee E, Schermitzler B, Leipold D, Sponheim S (2021). Disturbances in aperiodic neural activity during resting state in patients with schizophrenia. Biol Psychiatry.

[26] Gao R, Peterson EJ, Voytek B (2017). Inferring synaptic excitation/inhibition balance from field potentials. Neuroimage.

[27] Mandali A, Manssuer L, Zhao Y, Zhang C, Wang L, Ding Q (2021). Acute time-locked alpha frequency subthalamic stimulation reduces negative emotional bias in Parkinson’s disease. Biol Psychiatry Cogn Neurosci Neuroimaging.

[28] Huang Y, Sun B, Debarros J, Zhang C, Zhan S, Li D (2021). Increased theta/alpha synchrony in the habenula-prefrontal network with negative emotional stimuli in human patients. Elife.

[29] He N, Sethi SK, Zhang C, Li Y, Chen Y, Sun B (2020). Visualizing the lateral habenula using susceptibility weighted imaging and quantitative susceptibility mapping. Magn Reson imaging.

[30] Wang Y, Zhang C, Zhang Y, Gong H, Li J, Jin H (2020). Habenula deep brain stimulation for intractable schizophrenia: a pilot study. Neurosurgical Focus.

[31] Horn A, Kühn AA (2015). Lead-DBS: a toolbox for deep brain stimulation electrode localizations and visualizations. Neuroimage.

[32] Avants BB, Tustison NJ, Song G, Cook PA, Klein A, Gee JC (2011). A reproducible evaluation of ANTs similarity metric performance in brain image registration. Neuroimage.

[33] Fonov V, Evans AC, Botteron K, Almli CR, McKinstry RC, Collins DL (2011). Unbiased average age-appropriate atlases for pediatric studies. Neuroimage.

[34] Husch A, Petersen MV, Gemmar P, Goncalves J, Hertel F (2018). PaCER-A fully automated method for electrode trajectory and contact reconstruction in deep brain stimulation. NeuroImage: Clin.

[35] Delorme A, Makeig S (2004). EEGLAB: an open source toolbox for analysis of single-trial EEG dynamics including independent component analysis. J Neurosci Methods.

[36] Lang PJ, Bradley MM, Cuthbert BN (1997). International affective picture system (IAPS): Technical manual and affective ratings. NIMH Cent Study Emot Atten.

[37] Makeig S (1993). Auditory event-related dynamics of the EEG spectrum and effects of exposure to tones. Electroencephalogr Clin Neurophysiol.

[38] Grandchamp R, Delorme A (2011). Single-trial normalization for event-related spectral decomposition reduces sensitivity to noisy trials. Front Psychol.

[39] Maris E, Oostenveld R (2007). Nonparametric statistical testing of EEG-and MEG-data. J Neurosci Methods.

[40] Huebl J, Brücke C, Merkl A, Bajbouj M, Schneider G-H, Kühn AA (2016). Processing of emotional stimuli is reflected by modulations of beta band activity in the subgenual anterior cingulate cortex in patients with treatment resistant depression. Soc Cogn Affect Neurosci.

[41] Huebl J, Schoenecker T, Siegert S, Brücke C, Schneider GH, Kupsch A (2011). Modulation of subthalamic alpha activity to emotional stimuli correlates with depressive symptoms in Parkinson’s disease 1. Mov Disord.

[42] Medel V, Irani M, Ossandon T and Boncompte G. Complexity and 1/f slope jointly reflect cortical states across different E/I balances. Preprint at https://www.biorxiv.org/content/10.1101/2020.09.15.298497v1 (2020).

[43] Waschke L, Tune S, Obleser J (2019). Local cortical desynchronization and pupil-linked arousal differentially shape brain states for optimal sensory performance. Elife.

[44] Pfeffer T, Avramiea A-E, Nolte G, Engel AK, Linkenkaer-Hansen K, Donner TH (2018). Catecholamines alter the intrinsic variability of cortical population activity and perception. PLoS Biol.

[45] Dosenbach NU, Fair DA, Miezin FM, Cohen AL, Wenger KK, Dosenbach RA, (2007). Distinct brain networks for adaptive and stable task control in humans. Proc Natl Acad Sci USA.

[46] Lee U, Oh G, Kim S, Noh G, Choi B, Mashour GA (2010). Brain networks maintain a scale-free organization across consciousness, anesthesia, and recovery: evidence for adaptive reconfiguration. J Am Soc Anesthesiologists.

[47] Concha ML, Ahumada-Galleguillos P (2016). An evolutionary perspective on habenular asymmetry in humans. J Neurol Neuromed.

[48] Sohal VS, Rubenstein JL (2019). Excitation-inhibition balance as a framework for investigating mechanisms in neuropsychiatric disorders. Mol psychiatry.

[49] Morris J, Smith K, Cowen P, Friston K, Dolan RJ (1999). Covariation of activity in habenula and dorsal raphe nuclei following tryptophan depletion. Neuroimage.

[50] Caldecott-Hazard S, Mazziotta J, Phelps M (1988). Cerebral correlates of depressed behavior in rats, visualized using 14C-2-deoxyglucose autoradiography. J Neurosci.

[51] Berman RM, Cappiello A, Anand A, Oren DA, Heninger GR, Charney DS (2000). Antidepressant effects of ketamine in depressed patients. Biol Psychiatry.

[52] Murrough JW, Iosifescu DV, Chang LC, Al Jurdi RK, Green CE, Perez AM (2013). Antidepressant efficacy of ketamine in treatment-resistant major depression: a two-site randomized controlled trial. Am J Psychiatry.

[53] Hauptman JS, DeSalles AA, Espinoza R, Sedrak M, Ishida W (2008). Potential surgical targets for deep brain stimulation in treatment-resistant depression. Neurosurgical focus.

[54] Park MR (1987). Monosynaptic inhibitory postsynaptic potentials from lateral habenula recorded in dorsal raphe neurons. Brain Res Bull.

[55] Reisine TD, Soubrie P, Artaud F, Glowinski J (1982). Involvement of lateral habenula-dorsal raphe neurons in the differential regulation of striatal and nigral serotonergic transmission cats. J Neurosci.

[56] Wang RY, Aghajanian GK (1977). Physiological evidence for habenula as major link between forebrain and midbrain raphe. Science.

[57] Stern W, Johnson A, Bronzino J, Morgane P (1979). Effects of electrical stimulation of the lateral habenula on single-unit activity of raphe neurons. Exp Neurol.

[58] Fraschini M, Meli M, Demuru M, Didaci L, Barberini L (2020). EEG fingerprints under naturalistic viewing using a portable device. Sensors.

[59] Walker CP, Buse JB, Frohlich F (2021). Experimental increase of blood glucose alters resting state EEG measures of excitation–inhibition balance. Exp Physiol.

[60] Foxe JJ, Snyder AC (2011). The role of alpha-band brain oscillations as a sensory suppression mechanism during selective attention. Front Psychol.

[61] Pfurtscheller G, Aranibar A (1977). Event-related cortical desynchronization detected by power measurements of scalp EEG. Electroencephalogr Clin Neurophysiol.

[62] Huebl J, Spitzer B, Brücke C, Schönecker T, Kupsch A, Alesch F (2014). Oscillatory subthalamic nucleus activity is modulated by dopamine during emotional processing in Parkinson’s disease. Cortex.

[63] Kühn A, Hariz M, Silberstein P, Tisch S, Kupsch A, Schneider G-H (2005). Activation of the subthalamic region during emotional processing in Parkinson disease. Neurology.

[64] Kemp AH, Gray MA, Eide P, Silberstein R, Nathan PJ (2002). Steady-state visually evoked potential topography during processing of emotional valence in healthy subjects. NeuroImage.

[65] Popov T, Steffen A, Weisz N, Miller GA, Rockstroh B (2012). Cross‐frequency dynamics of neuromagnetic oscillatory activity: two mechanisms of emotion regulation. Psychophysiology.

[66] Gotlib IH, Krasnoperova E, Yue DN, Joormann J (2004). Attentional biases for negative interpersonal stimuli in clinical depression. J Abnorm Psychol.

[67] Davis H (1968). Slow electrical responses of the human cortex. Proc Am Philos Soc.

[68] Demanuele C, James CJ, Sonuga-Barke EJ (2007). Distinguishing low frequency oscillations within the 1/f spectral behaviour of electromagnetic brain signals. Behav Brain Funct.

[69] Gustafson DE, Eterno JS and Jarisch W. Signal analysis techniques for interpreting electroencephalograms. 1980, Scientific Systems Inc Cambridge MA.

[70] Linkenkaer-Hansen K, Nikouline VV, Palva JM, Ilmoniemi RJ (2001). Long-range temporal correlations and scaling behavior in human brain oscillations. J Neurosci.

[71] Van de Ville D, Britz J, Michel CM (2010). EEG microstate sequences in healthy humans at rest reveal scale-free dynamics. Proc Natl Acad Sci.

[72] Grüneis F, Nakao M, Yamamoto M, Musha T, Nakahama H (1989). An interpretation of 1/f fluctuations in neuronal spike trains during dream sleep. Biol Cybern.

[73] Miller KJ, Sorensen LB, Ojemann JG, Den M (2009). Nijs, Power-law scaling in the brain surface electric potential. PLoS Comput Biol.

[74] Browne CA, Hammack R, Lucki I (2018). Dysregulation of the lateral habenula in major depressive disorder. Front Synaptic Neurosci.

[75] Graziane NM, Neumann PA, Dong Y (2018). A focus on reward prediction and the lateral habenula: functional alterations and the behavioral outcomes induced by drugs of abuse. Front Synaptic Neurosci.

[76] Proulx CD, Hikosaka O, Malinow R (2014). Reward processing by the lateral habenula in normal and depressive behaviors. Nat Neurosci.

