# Supplementary material for: Power signatures of habenular neuronal signals in patients with bipolar or unipolar depressive disorders correlate with their disease severity
Source: Transl Psychiatry. 2022 Feb 22;12:72. doi: 10.1038/s41398-022-01830-3 (PMC8863838; doi:10.1038/s41398-022-01830-3)
Supplement: Supplementary file 1 — Supplementary Material [file 41398_2022_1830_MOESM1_ESM.docx]

**Supplementary Material**

**Power signatures of habenular neuronal signals in patients with bipolar or unipolar depressive disorders correlate with their disease severity**

Saurabh Sonkusare, Qiong Ding, Yingying Zhang, Linbin Wang, Hengfen Gong Alekhya Mandali, Luis Manssuer, Yijie Zhao, Yixin Pan, Chencheng Zhang, Dianyou Li, Bomin Sun, and Valerie Voon

STable 1: Mean valence ratings by the patients

| P ID | Valence Rating (0-100) | | |
| --- | --- | --- | --- |
|  | Positive | Neutral | Negative |
| 1 | 55.2 | 50.7 | 45.5 |
| 3 | 74.9 | 59.7 | 26.5 |
| 4 | 66.8 | 59.8 | 15.3 |
| 5 | 60.1 | 50.7 | 18.4 |
| 6 | 62.7 | 47.8 | 14.5 |

^a^ valence ratings could not be undertaken by P2


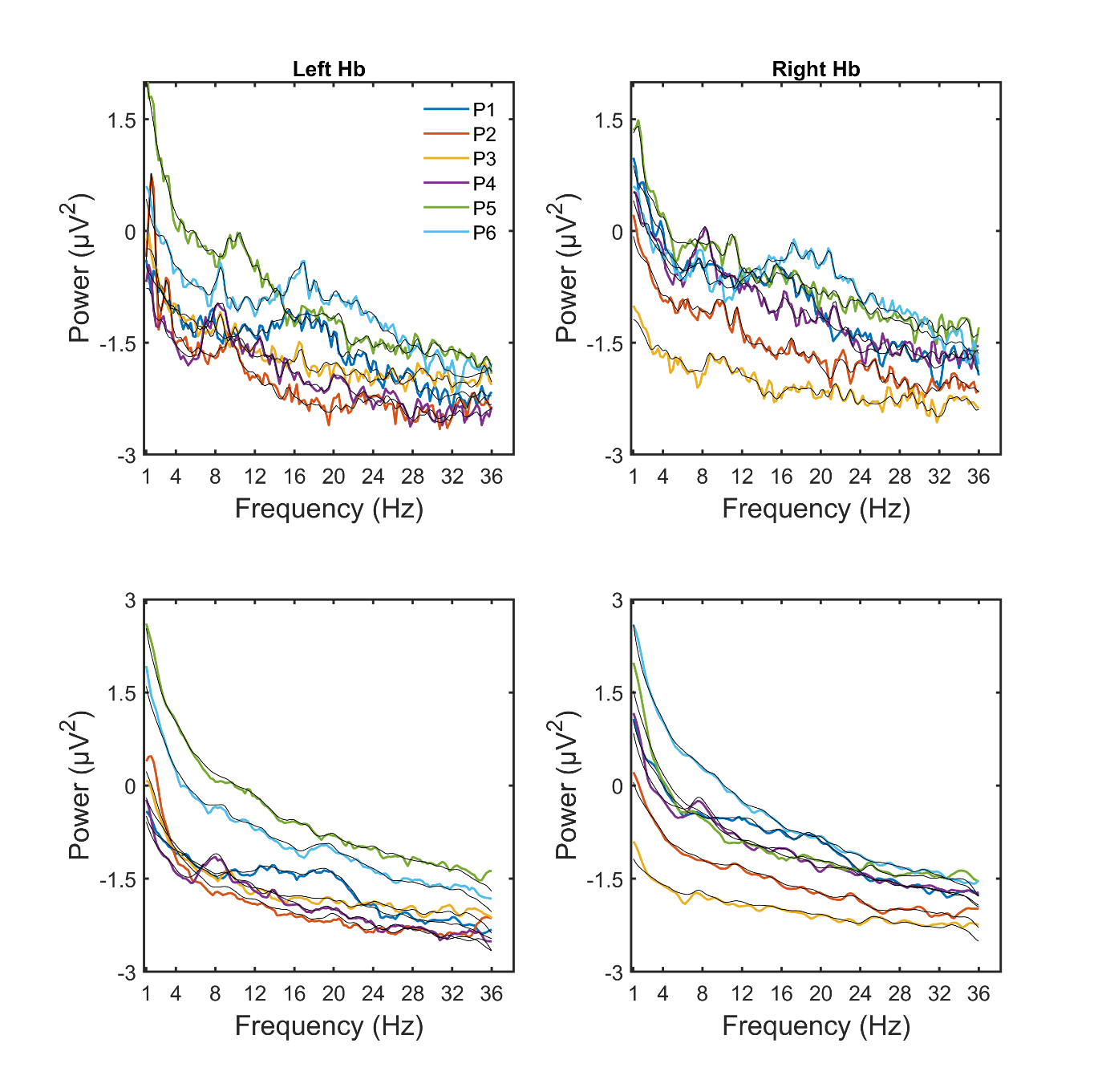


**SFig1. Individual patient’s power profile.** Power profile for resting state (top) and task (bottom). Power fit via *fooof* algorithm is shown in black lines.


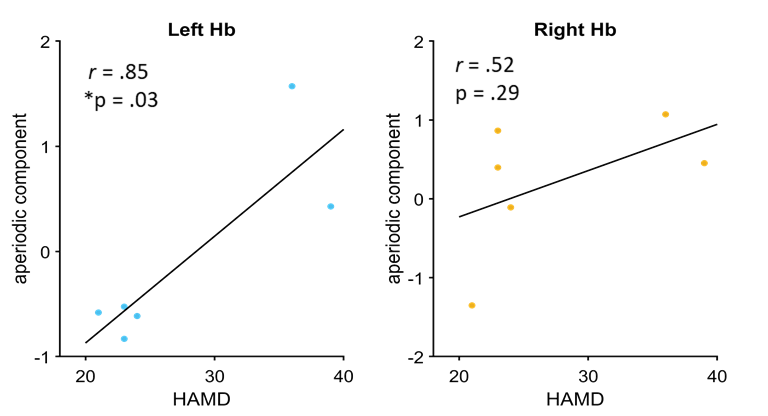


**SFig2. Association of aperiodic component computed with 1-40 Hz frequency range with depression scores.** We replicate the significant association between aperiodic component of left habenula and depression scores.


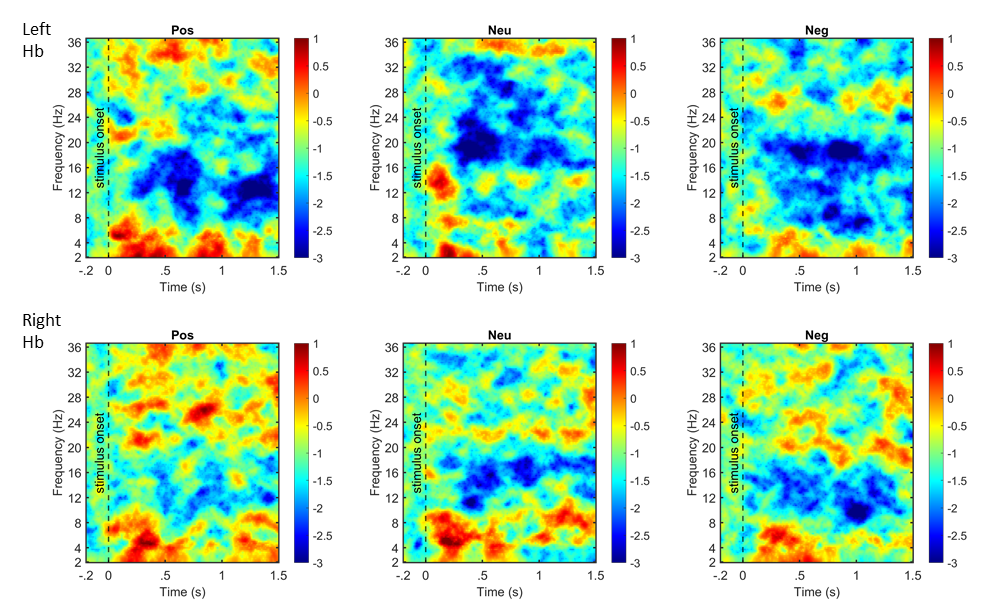


**SFig3.** **Stimulus locked time frequency responses of habenula (Hb).** Event related spectral perturbation (ERSP) maps for three valence conditions – positive (Pos), neutral (Neu) and negative (Neg). Event related desynchronization (ERD) is observed for all types of stimuli especially in alpha and beta frequency range.


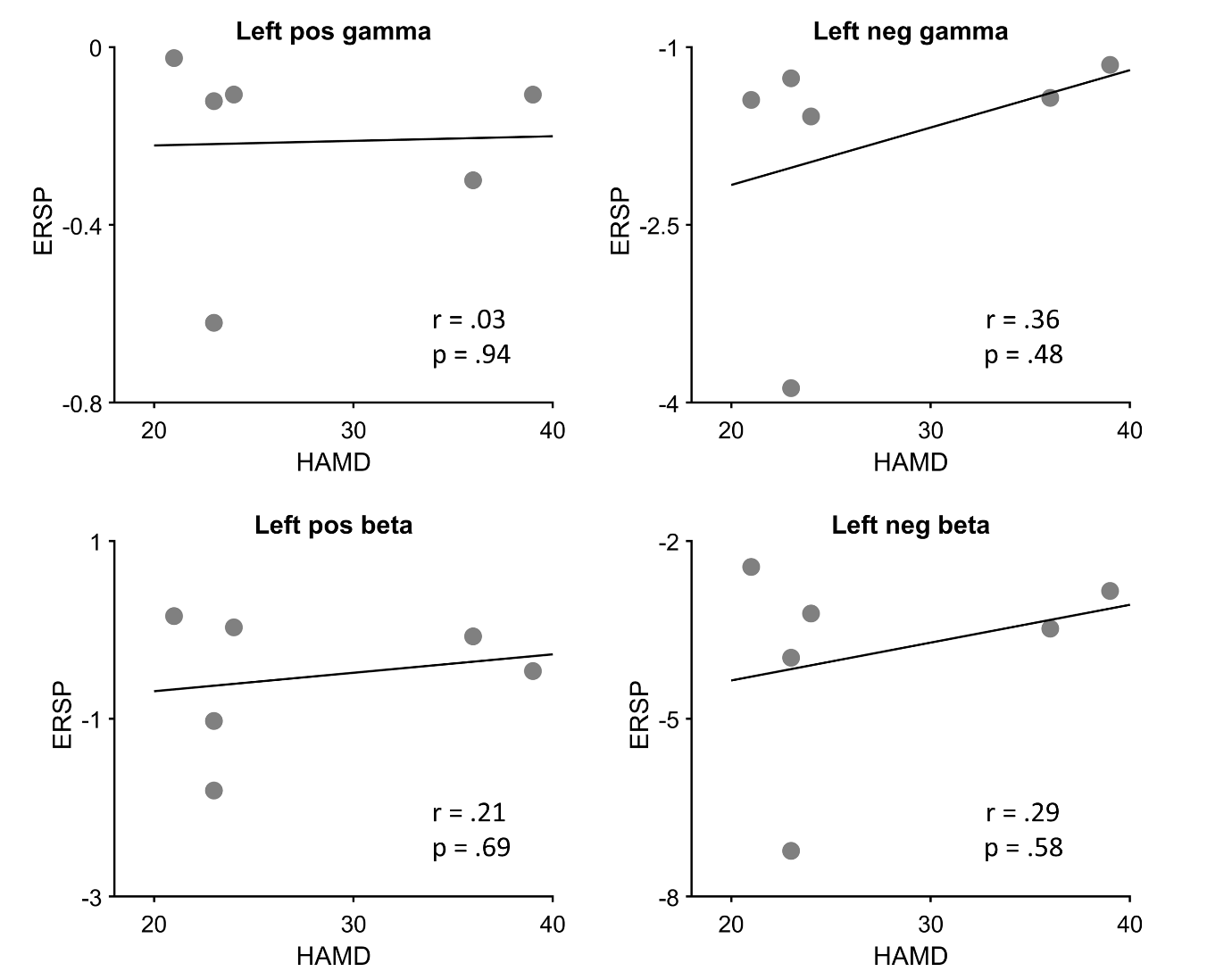


**SFig4. Left habenula task condition difference activity correlation with depression severity.** Top panel: No significant correlation gamma cluster activity for positive stimuli (left) and negative stimuli (right) with Hamilton Depression rating scale (HAMD); bottom panel: No significant correlation beta cluster activity for positive stimuli (left) and negative stimuli (right) with Hamilton Depression rating scale (HAMD). Pos – positive condition, neg – negative condition.


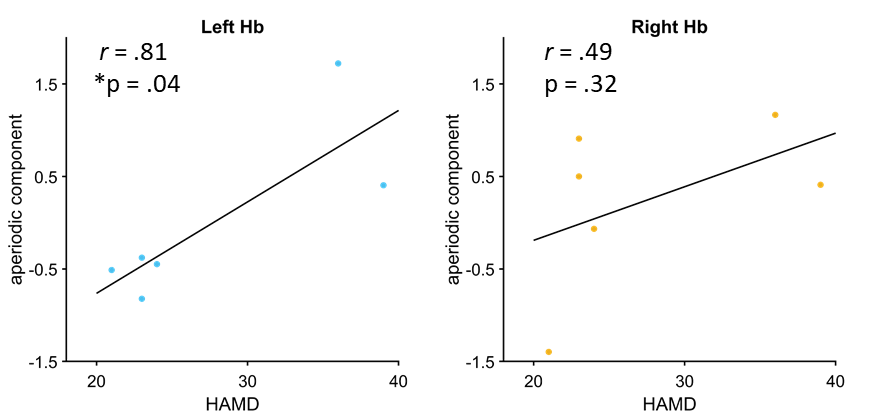


**SFig5. Association of aperiodic component computed on short resting state epochs with depression scores.** The aperiodic component was computed on 2.5 second epochs of resting state data. Consistent association of aperiodic component of left habenula signals with depression scores was observed.

**Prefrontal-habenula connectivity**

The functional connectivity between habenula and prefrontal scalp EEG was investigated using coherence analysis, which provides a frequency domain measure of the degree of co-variability between signals (van Wijk et al., 2017). We followed the procedure described by Boonstra et al 2007 (Boonstra, Daffertshofer, Breakspear, & Beek, 2007) to estimate coherence with a frequency resolution of .25 and temporal resolution of 20ms. First, complex valued coherency was estimated for all the trials (-500ms to 1500ms) within each condition for each participant. The absolute value of coherency was squared to obtain time varying magnitude-squared coherence. These time varying coherence maps were then used for statistical testing of differences via permutation testing as outlined in the main text.


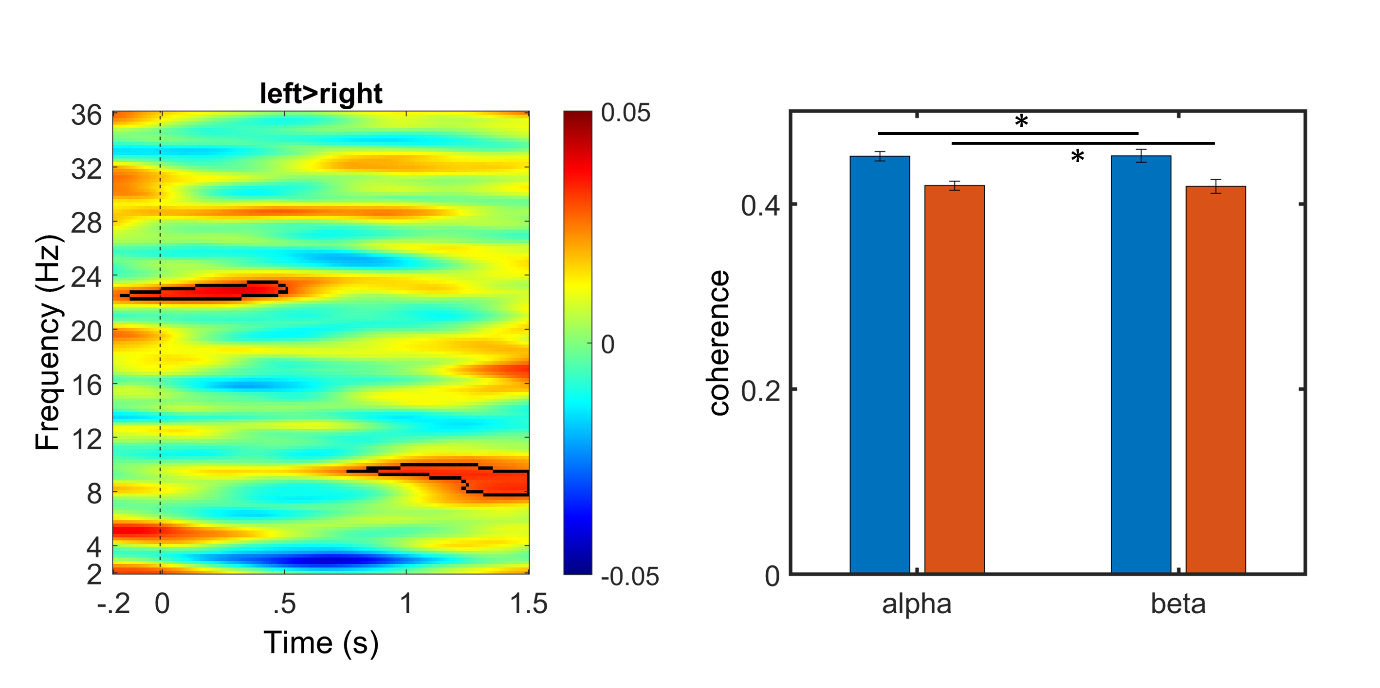


**SFig6.** *Coherence between habenula and prefrontal cortex*. Left: contrast of time varying coherence between left and right habenula with prefrontal cortex for all trials of the conditions concatenated and statistically tested via permutation testing. Significant cluster shown in black outlines. Vertical line at zero indicates stimulus onset. Right: bar plot showing the mean coherence values from the significant clusters (left habenula in blue, right habenula in red) (alpha: mean left = .45, SD = .15, mean right = .42, SD = .14; *t(443)=4.78, P=0.0006,* (beta: mean left = .45, SD = .14, mean right= .41, SD = .15; *t(443)=3.3, P=0.001*). Error bars showing standard error of mean. *Bonferroni correction P<.025.

**References**

Boonstra, T. W., Daffertshofer, A., Breakspear, M., & Beek, P. J. (2007). Multivariate time–frequency analysis of electromagnetic brain activity during bimanual motor learning. *Neuroimage, 36*(2), 370-377.

van Wijk, B. C., Neumann, W.-J., Schneider, G.-H., Sander, T. H., Litvak, V., & Kühn, A. A. (2017). Low-beta cortico-pallidal coherence decreases during movement and correlates with overall reaction time. *Neuroimage, 159*, 1-8.
